# Empirical antibiotic therapy for pneumonia in intensive care units: a multicentre, retrospective analysis of potentially pathogenic microorganisms identified by endotracheal aspirates cultures

**DOI:** 10.1007/s10096-015-2482-y

**Published:** 2015-09-18

**Authors:** J. B. J. Scholte, H. L. Duong, C. Linssen, H. Van Dessel, D. Bergmans, R. van der Horst, P. Savelkoul, P. Roekaerts, W. van Mook

**Affiliations:** Zentrum für Intensivmedizin, Luzerner Kantonsspital, 6000 Luzern 16, Switzerland; Department of Intensive Care Medicine, Maastricht University Medical Centre+, Maastricht, The Netherlands; Department of Medical Microbiology, Zuyderland Medical Centre, Heerlen, The Netherlands; Department of Medical Microbiology, Maastricht University Medical Centre+, Maastricht, The Netherlands; Department of Pulmonary Medicine and Intensive Care Medicine, Zuyderland Medical Centre, Heerlen, The Netherlands

## Abstract

The purpose of this investigation was to explore the presumed relationship between the days of hospitalisation and microorganisms identified by endotracheal aspirate cultures in relation to adequate empirical treatment strategies of pneumonia in the intensive care unit (ICU). All potentially pathogenic microorganisms identified by (surveillance) cultures of endotracheal aspirates obtained in the ICUs of two Dutch teaching hospitals in 2007 and 2012 were retrospectively collected and analysed. Antibiotic susceptibilities to 11 antibiotics were calculated for several time points (days or weeks) after hospital admission and expressed per patient-day. In total, 4184 potentially pathogenic microorganisms identified in 782 patients were analysed. Prevalence of the classic early-onset pneumonia-causing microorganisms decreased from 55 % on the first four days to 34 % on days 4–6 after hospital admission (*p* < 0.0001). Susceptibility to amoxicillin/clavulanic acid was below 70 % on all days. Except for days 0 and 12, susceptibility to ceftriaxone was below 80 %. The overall susceptibility to piperacillin/tazobactam was 1518/1973 (77 %) in 2007 vs. 727/1008 (67 %) in 2012 (*p* < 0.0001). After day 8 of hospital admission, susceptibility to piperacillin/tazobactam therapy was below 80 % in 2012. After one week of hospital admission, susceptibilities to antibiotics were lower in the hospital that included that antibiotic in the local empirical treatment protocols as compared to the hospitals in which that antibiotic was not or infrequently included: 90/434 (21 %) vs. 117/398 (29 %); *p* = 0.004 for amoxicillin/clavulanic acid and 203/433 (47 %) vs. 253/398 (64 %); *p* < 0.001 for ceftriaxone. No cut-off in the number of days after hospital admission could be identified to distinguish early-onset from late-onset pneumonia. Consequently, the choice of empirical antibiotics should probably not be based on the time of onset.

**Electronic supplementary material:**

The online version of this article (doi:10.1007/s10096-015-2482-y) contains supplementary material, which is available to authorized users.

## Introduction

Pneumonia in the intensive care unit (ICU) is associated with significant mortality, morbidity and costs [1, 2]. Early and appropriate antibiotic treatment reduces pneumonia mortality [3], but unnecessary and inappropriate antibiotic use leads to redundant side effects, costs and promotes antibiotic resistance [4, 5]. Selecting the most optimal empirical antibiotic treatment of pneumonia in ICUs should depend on many factors, such as suspected causative microorganism, local resistance profiles, specific pharmacological and pharmacodynamic characteristics, local protocols and antibiotic costs. A specific number of hospital admission days prior to pneumonia development is frequently used as a cut-off after which antibiotic therapy is switched in order to target more multidrug-resistant (MDR) and hospital-acquired microorganisms [6]. For ventilator-associated pneumonia (VAP), the distinction between early-onset and late-onset VAP is, therefore, frequently made. However, the cut-off in the number of days to distinguish late-onset from early-onset VAP is used inconsistently. In 1987, late-onset VAP was defined as a pneumonia that developed after 5 days of ICU admission [7], whereas 5 days [8] or 7 days [9, 10] after the start of mechanical ventilation are likewise used. Studies using 5 days after intubation or 4–7 days after ICU admission as the cut-off value demonstrated no microbiological differences in the prevalence of potential MDR microorganisms between both groups [8, 11]. Indeed, a cut-off of 5 days after hospital admission may be more rational, since patients who are hospitalised for five or more days before intubation will harbour microorganisms more commonly associated with late-onset pneumonia [6, 12].

Potentially pathogenic microorganisms previously identified by (surveillance) cultures of endotracheal aspirates (ETAs) are, regardless of quantification, known to be frequent causes of subsequently developed VAP [13]. Indeed, the diagnosis of VAP is frequently confirmed by these ETA cultures [6, 14, 15]. Additionally, antibiotic guidance by surveillance cultures in a hypothetic model of 223 hospital-acquired pneumonia patients resulted in the use of a smaller number of broad-spectrum antibiotics without reducing appropriateness [16]. Because clinicians aim to target these previously identified microorganisms in the case where pneumonia develops [17], one could hypothesise that the results of antibiotic susceptibility of these ETA cultures could be useful to analyse the general appropriateness of empirical antibiotics.

In the perspective of this assumption, the current study aims to explore whether a cut-off value in days after hospital admission can be identified which best distinguishes early- from late-onset pneumonia in the ICU. This study also provides insight into the most appropriate empirical treatment depending on the time after admission. Furthermore, differences between two different years and hospitals concerning MDR development are appraised.

## Methods

### Setting

The study was conducted in two medical centres in the Netherlands (known for relatively low MDR rates [18]); the Maastricht University Medical Centre, a 715-bed tertiary university medical centre with approximately 30,000 admissions annually and 27 ICU beds (further referred to as hospital A), and the Zuyderland Medical Centre Heerlen, a general 1230-bed teaching hospital (19 km from hospital A) with approximately 30,000 admissions annually and 21 ICU beds (hospital B). Surveillance cultures of ETA were obtained twice weekly from all mechanically ventilated patients in both hospitals. Furthermore, ETA cultures were performed under the suspicion of an infection. When pneumonia was present and no results of previously obtained cultures were available for antibiotic guidance, empirical treatment algorithms were different in the two hospitals. In hospital A, ICU patients that develop pneumonia within the first four days of hospital admittance receive amoxicillin/clavulanic acid frequently with ciprofloxacin and piperacillin/tazobactam when admitted to the ICU after four preceding days of admission to a hospital ward. In hospital B, ICU patients that developed pneumonia within the first four days of hospital admittance receive either amoxicillin/clavulanic acid or ceftriaxone including ciprofloxacin. When the pneumonia developed after four days of hospital admittance, piperacillin/tazobactam is usually administered. In both hospitals, the ciprofloxacin is stopped when the patient recovers and the urine Legionella antigen test is negative. The attendance of a consulting medical microbiologist in daily multidisciplinary ICU meetings contributes to good antibiotic stewardship.

In hospital A, selective oropharyngeal decontamination (SOD) use was implemented in December 2010 and selective digestive tract decontamination (SDD), which includes SOD, was implemented after January 2012 in patients expected to stay in the ICU for more than 48 h. In 2012, hospital B used oropharyngeal decontamination with chlorhexidine. In 2007, the hospitals used neither SOD nor SDD. The ethics committees of both the institutions approved the study. Informed consent was not necessary, since it concerned a retrospective study evaluating standard patient care and patients’ privacy was respected.

### Design and definitions

All potentially pathogenic microorganisms, including their antibiotic susceptibility to 11 frequently used antibiotics, identified by (surveillance) cultures of ETA obtained in 2007 and 2012 were retrospectively collected. In hospital A, the data query was used from a Phoenix automated microbiology system for susceptibility testing (DB Diagnostics, Sparks, MD, USA) and from the laboratory information system (Labosys, Philips, Eindhoven, The Netherlands). In hospital B, the data query was performed using VITEK 2 (automated system for microorganisms identification and antibiotic susceptibility testing; bioMérieux, Inc., Durham, NC, USA). Endotracheal aspirates were cultured semiquantitatively and growth was expressed as sporadic, little, moderate or heavy. As no threshold for positivity is known for semiquantitative cultures of ETA, all positive results were included. Clinical data were received from patient data management systems. In case multiple identical microorganisms with identical antibiotic susceptibility were identified on the same day in the same patient, only one microorganism was included in the susceptibility analysis. The included microorganisms were subsequently arranged according to the number of days after hospital admission the ETA sample was collected. Subsequently, antibiotic susceptibility on a specific day after hospital admission was calculated and expressed per patient-day. The latter signifies that, in case multiple microorganisms were identified in the same patient on the same day after hospital admission, susceptibility to a specific antibiotic was defined only when all microorganisms identified on a specific day after hospital admission were susceptible to that antibiotic. All ETA cultures were analysed regardless of being surveillance cultures or cultures taken for suspected pneumonia, and regardless of the presence or absence of infection. Based on the elucidation in the introduction, antibiotic appropriateness may, thus, be equal to the calculated susceptibility of all microorganisms identified that day in one patient to a specific antibiotic. Consequently, one can extrapolate the appropriateness of different empirical antibiotics on any day after hospital admission a pneumonia (hypothetically) presents. The classical early-onset VAP microorganisms are *Streptococcus pneumoniae*, *Haemophilus influenzae*, *Moraxella catarrhalis* and (methicillin-sensitive) *Staphylococcus aureus*. By comparing antibiotic susceptibility between different days after hospital admission, the most rational cut-off in days for early- and late-onset VAP and, consequently, antibiotic change can be identified. A meta-analysis of 87 studies reported a pooled rate of appropriate empirical antibiotic treatment of 71.4 % [95 % confidence interval (CI) 68.2–74.6] [19]. Additional literature concerning the optimal and/or acceptable cut-off in the number of days to strive for is currently lacking. Therefore, hypothetically, antibiotic appropriateness was arbitrarily defined as follows: at least 80 % of the microorganisms identified on a particular day (per patient) after hospital admission should be susceptible to the appraised antibiotic. In order to determine whether differences in susceptibility arise due to differences in empirical antibiotic use, antibiotic susceptibilities after one week of hospitalisation were compared between the hospitals.

### Antibiotic susceptibility

The susceptibility patterns of the following antibiotics were collected: amoxicillin, amoxicillin/clavulanic acid, ceftazidime, ceftriaxone, ciprofloxacin, gentamicin, meropenem, piperacillin, piperacillin/tazobactam, vancomycin and trimethoprim/sulphamethoxazole. The antibiotic susceptibility of all samples obtained in 2007 were adjusted to European Committee on Antimicrobial Susceptibility Testing (EUCAST) v 2.0 guidelines (introduced in 2010 [20]) to ensure their comparability to the 2012 cohort. When susceptibility was not tested and the EUCAST did not provide presumed susceptibility, suppositions were made in the following cases (partly based on the low–mediocre MDR situation in the Netherlands [21]):

- *Moraxella catarrhalis* was considered susceptible to ciprofloxacin.
- *Streptococcus pneumoniae* was considered susceptible to meropenem and resistant to ceftazidime.
- Enterobacteriaceae and *M. catarrhalis* susceptible to amoxicillin were presumed to be susceptible to piperacillin and, if susceptible to amoxicillin/clavulanic acid, susceptibility to piperacillin/tazobactam was presumed.
- When *Pseudomonas aeruginosa* was susceptible to imipenem, susceptibility to meropenem was presumed.
- Susceptibility of *H. influenzae*, *S. pneumoniae* and *M. catarrhalis* to gentamicin was neither tested nor known. Therefore, these microorganisms were excluded in the susceptibility testing of gentamicin and excluded from the calculations.
- In case of methicillin-resistant *S. aureus* (MRSA), resistance to meropenem and piperacillin/tazobactam was not tested by standard practice and was, therefore, deduced.

In hospital B, susceptibility testing of Enterobacteriaceae to piperacillin was not routinely performed and, consequently, the appropriateness of piperacillin therapy was not speculated upon.

### Exclusion criteria

Commensal flora (*Candida* spp., coagulase-negative staphylococci, *Corynebacterium* spp., enterococci, *Neisseria* spp., oropharyngeal flora, *Elizabethkingia meningoseptica*, *Streptococcus viridians* group and yeasts) were considered non-pathogenic and, subsequently, these organisms were not included, consistent with previous studies and guidelines [22–24]. *Stenotrophomonas maltophilia* was excluded from the antibiotic susceptibility analysis due to high intrinsic resistance rates, whereas pathogenicity is considered to be limited [25, 26]. *Sphingomonas paucimobilis*, *Alcaligenes xylosoxidans*, *Pseudomonas putida* and miscellaneous non-fermenters were rarely identified and were excluded from the susceptibility analysis.

### Statistics

An associate professor in statistics advised regarding the data analysis. Numbers are presented as the mean including standard deviation when appropriate. Percentages are given as integers. Due to the fact that more microorganisms than patients were included, differences in microorganism prevalence between the two hospitals and years were expressed in corrected odds ratio (COR), using generalised estimating equations correcting for microorganisms that are repeatedly cultured in the same patient. Differences in incidences of a categorical variable between two groups were calculated using the Pearson Chi-square test. In order to define the best cut-off point in days after hospital admission to differentiate early-onset VAP from late-onset VAP, the most clear and permanent drop in antibiotic susceptibility between two consecutive days after hospital admission was visualised and subsequently used. IBM SPSS Statistics version 23 for Windows (Chicago, IL, USA) was used for the analyses.

## Results

### General findings

From the 6524 ETA samples obtained during the studied period, 4184 potentially pathogenic microorganisms from 782 patients were identified. Table 1 provides information regarding general patient characteristics, indications for ICU admission and the bacteria identified. In both hospitals, more potentially pathogenic microorganisms were identified in 2007 compared to 2012 (total 2643 vs. 1541).

**Table 1:** Patient characteristics and endotracheal aspirates culture results

	Hospital A		Hospital B		Total
Year	2007	2012	2007	2012	2007 and 2012
ICU admissions	1688	1807	1347	1271	6113
Samples obtained	1759	1550	1849	1366	6524
PPMO identified	1595	816	1048	725	4184
Patients	234	194	194	160	782
Mean age, years (SD)	63 (15)	61 (14)	68 (12)	65 (14)	64 (14)
Male (%)	147 (63)	137 (71)	126 (65)	105 (66)	515 (66)
Indication for ICU admission (% of patients)^a^					
Non-surgical	81 (35 %)	105 (54 %)	120 (62 %)	105 (66 %)	411 (53 %)
Respiratory	43 (18 %)	34 (18 %)	43 (22 %)	51 (32 %)	171 (22 %)
Neurological	12 (5 %)	20 (10 %)	22 (11 %)	15 (9 %)	69 (9 %)
Post cardiac arrest	4 (2 %)	16 (8 %)	15 (8 %)	19 (12 %)	54 (7 %)
Cardiovascular	6 (3 %)	8 (4 %)	19 (10 %)	12 (8 %)	45 (6 %)
Abdominal	6 (3 %)	6 (3 %)	10 (5 %)	5 (3 %)	27 (3 %)
Surgical	153 (65 %)	89 (46 %)	74 (38 %)	55 (34 %)	371 (47 %)
Cardiovascular	58 (25 %)	42 (22 %)	15 (8 %)	5 (3 %)	120 (15 %)
Abdominal	31 (13 %)	12 (6 %)	43 (22 %)	30 (19 %)	116 (15 %)
Trauma	20 (9 %)	12 (6 %)	6 (5 %)	6 (4 %)	44 (6 %)
Neurological	24 (10 %)	15 (8 %)	2 (1 %)	2 (1 %)	43 (5 %)
Bacteria identified (% of total PPMO identified)					
Gram-positive	147 (9 %)	85 (10 %)	222 (21 %)	157 (22 %)	611 (15 %)
*Staphylococcus aureus*	126 (8 %)	75 (9 %)	201 (19 %)	138 (19 %)	540 (13 %)
Of which MRSA	4		9 (1 %)	9 (1 %)	22 (1 %)
*Streptococcus pneumoniae*	21 (1 %)	10 (1 %)	21 (2 %)	19 (3 %)	71 (2 %)
Gram-negative	1448 (91 %)	731 (90 %)	826 (79 %)	568 (78 %)	3573 (85 %)
Non-fermenters	689 (43 %)	308 (38 %)	428 (41 %)	182 (25 %)	1607 (38 %)
*Acinetobacter* spp.	122 (8 %)	32 (4 %)	17 (2 %)	6 (1 %)	177 (4 %)
*Moraxella catarrhalis*	6	5 (1 %)	22 (2 %)	10 (1 %)	43 (1 %)
*Pseudomonas aeruginosa*	520 (33 %)	159 (19 %)	342 (33 %)	126 (17 %)	1147 (27 %)
*Stenotrophomonas maltophilia*	37 (2 %)	99 (12 %)	47 (4 %)	36 (5 %)	219 (5 %)
Other non-fermenters (e.g. *S. paucimobilis*, *A. xylosoxidans*)	4	13 (2 %)		4 (1 %)	21 (1 %)
Enterobacteriaceae	725 (45 %)	386 (47 %)	360 (34 %)	355 (49 %)	1826 (44 %)
*Citrobacter* spp.	18 (1 %)	9 (1 %)	11 (1 %)	13 (2 %)	51 (1 %)
*Enterobacter* spp.	112 (7 %)	54 (7 %)	46 (4 %)	32 (4 %)	244 (6 %)
*Escherichia coli*	217 (14 %)	104 (13 %)	110 (10 %)	80 (11 %)	511 (12 %)
*Klebsiella* spp.	191 (12 %)	56 (7 %)	91 (9 %)	86 (12 %)	424 (10 %)
*Morganella morganii*	17 (1 %)	1	21 (2 %)	14 (2 %)	53 (1 %)
*Proteus* spp.	45 (3 %)	48 (6 %)	46 (4 %)	51 (7 %)	190 (5 %)
*Serratia* spp.	120 (8 %)	109 (13 %)	20 (2 %)	75 (10 %)	324 (8 %)
Other Enterobacteriaceae (e.g. *Hafnia alvei*, *Kluyvera* spp., *Raoultella planticola*)	5	5 (1 %)	15	4 (1 %)	29 (1 %)
Miscellaneous					
*Haemophilus (para-)influenzae*	34 (2 %)	36 (4 %)	36 (3 %)	31 (4 %)	137 (3 %)
Other species		1	2		3
Included for prevalence and susceptibility analysis					
Patients	230 (98 %)	181 (93 %)	187 (96 %)	154 (96 %)	752 (96 %)
Microorganisms	1554 (97 %)	704 (86 %)	1001 (96 %)	689 (95 %)	3948 (94 %)

If no numbers and/or percentages are provided, the incidences and/or percentages are equal to zero

*ICU* Intensive care unit; *MRSA* methicillin-resistant *Staphylococcus aureus*; *PPMO* potentially pathogenic microorganisms; *SD* standard deviation

^a^Infrequent indications for ICU admission were not incorporated

The most common indications for ICU admission were respiratory failure (22 %), post cardiovascular surgery and abdominal surgery (both 15 %). In hospital A, cardiovascular surgery and neurological surgery were more frequently the reasons for ICU admission, as compared to hospital B (23 % vs. 6 % [*p* < 0.001] and 9 % vs. 1 % [*p* < 0.001], respectively). In hospital B, abdominal surgery was more frequently the reason for ICU admission compared to hospital A (21 % vs. 10 % [*p* < 0.001]).

### Identified microorganisms per study year and per hospital

Overall, *P. aeruginosa*, *S. aureus*, *Escherichia coli* and *Klebsiella* spp. were the most frequently identified microorganisms; 1147 (27 % of the total identified potentially pathogenic microorganisms), 540 (13 %), 511 (12 %) and 424 (10 %) times, respectively. All COR including 95 % CIs and *p*-values regarding the prevalence of microorganisms identified in 2012 vs. 2007 and in hospital B vs. hospital A are provided in Appendix 1.

*Pseudomonas aeruginosa* was less frequently identified in 2012 as compared to 2007; 285 vs. 862 times (COR 0.470 [95 % CI 0.262–0.843] *p* = 0.011). *Stenotrophomonas maltophilia*, *Proteus* spp., *Serratia* spp. and *H.* (*para*)*influenzae* were relatively more frequently isolated in 2012 as compared to 2007: 135 vs. 84 times (COR 2.988 [95 % CI 1.458–6.126] *p* = 0.003), 99 vs. 91 times (COR 1.881 [95 % CI 1.020–3.467] *p* = 0.043), 184 vs. 140 times (COR 2.563 [95 % CI 1.271–5.169] *p* = 0.009) and 67 vs. 70 times (COR 1.642 [1.027–2.626] *p* = 0.038), respectively.

*Staphylococcus aureus* was more frequently isolated in hospital B as compared to hospital A; 339 vs. 201 times (COR 2.589 [95 % CI 1.799–3.725] *p* < 0.001). Gram-negative microorganisms were significantly less frequently isolated in hospital B as compared to hospital A; 1394/1773 (79 %) vs. 2179/2411 (90 %) (COR 0.394 [95 % CI 0.279–0.555] *p* < 0.001). Both *M. catarrhalis* and *Morganella morganii* were more frequently isolated in hospital B as compared to hospital A: 32 times vs. 11 times (COR 4.062 [95 % CI 1.777–9.285] *p* = 0.001) and 35 vs. 19 times (COR 2.763 [95 % CI 1.120–5.816] *p* = 0.027). *Acinetobacter* spp. was less frequently isolated in hospital B as compared to hospital A; 33 vs. 154 times (COR 0.201 [95 % CI 0.076–0.526] *p* = 0.001).

### Prevalence of microorganisms according to the day after hospital admission

**Fig. 1:**
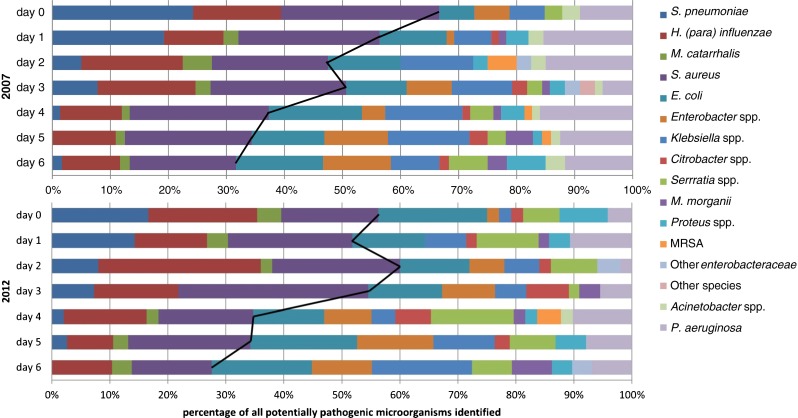
Prevalence of different microorganisms yielded by endotracheal aspirate samples in the first week of hospitalisation in 2007 and in 2012. The *black line* indicates the percentages of classical early-onset pneumonia pathogens (*Streptococcus pneumoniae*, *Haemophilus influenzae*, *Moraxella catarrhalis* and *Staphylococcus aureus*). *MRSA* Methicillin-resistant *Staphylococcus aureus*

A total of 3948 samples from 752 patients were included in the analyses concerning prevalence according to the day after hospital admission and antibiotic susceptibility. Figure 1 presents the prevalence of different microorganisms identified in ETA samples in the first week of hospital admission arranged according to the day after hospital admission in 2007 and 2012. A complete overview of the prevalence of specific microorganisms in the different years in specific days or weeks after hospital admission is provided in the Supplementary material. In Fig. 1, the black line indicates the percentage of classical early-onset pneumonia pathogens. In the first four days of admission, these microorganisms represent 55 % of all identified potentially pathogenic microorganisms (124/228 [54 %] in 2007 and 116/209 [56 %] in 2012), whereas in the last three days of the first week, these microorganisms represent 34 % (69/199 [35 %] in 2007 and 38/116 [33 %] in 2012; *p* < 0.0001 for comparing these different periods in both years). In 2007, *P. aeruginosa* represented 483/978 (49 %) of all microorganisms identified in ETA after week 5, whereas in 2012, *P. aeruginosa* rates increased every week to a steady 28 % (127/458) after week 3 (see Supplementary material). *Serratia* spp., which were more frequently identified in 2012 compared to 2007, represented 10–20 % of all potentially pathogenic microorganisms identified in the period after hospitalisation, although less in the first week (26/325 [8 %]) and significantly more after week 10 (30/133 [25 %], *p* < 0.001) in 2012 (see also Supplementary material).

### Antibiotic susceptibly testing

**Fig. 2:**
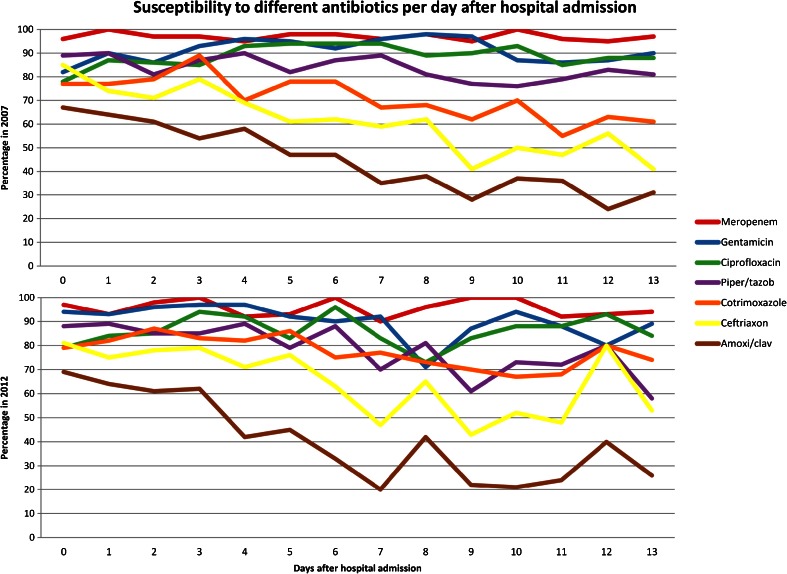
Susceptibility of all potentially pathogenic microorganisms yielded by endotracheal aspirates to different antibiotics in the first two weeks after hospital admission in 2007 and 2012

**Fig. 3:**
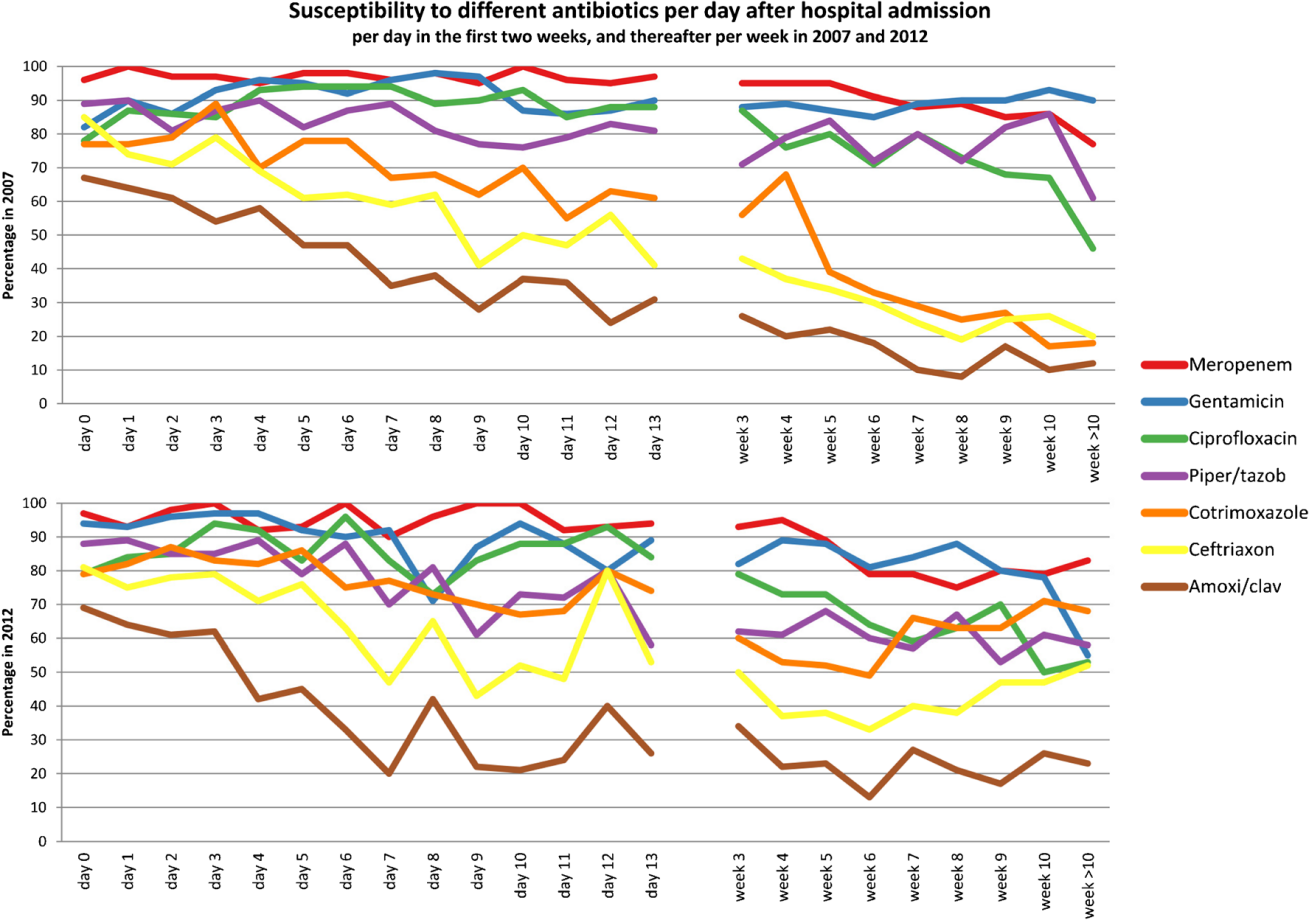
Antibiotic susceptibility of all potentially pathogenic microorganisms yielded by endotracheal aspirates arranged per patient-day in the first two weeks and per week thereafter

Antibiotic susceptibility testing results per patient-day in the first two weeks after hospital admission and the results arranged including the weeks thereafter are presented in Figs. 2 and 3. The presentation of the results is bisected in the two observed years. Looking at the first four days of hospital admission in 2012, susceptibility to amoxicillin/clavulanic acid was 104/164 (63 %), whereas on days 4–6, susceptibility revealed 37/91 (41 %) on days 4–6 (*p* = 0.0007). Indeed, in the transition from day 3 to day 4 of hospital admission, susceptibility dropped from 62 % (29/47) on day 3 to 42 % (16/38) on day 4 (*p* = 0.072).

Apart from a peak on day 12 (12/15 [80 %]), susceptibility to ceftriaxone remained below 80 % after the day of hospital admission. In 2012, more microorganisms yielded by ETA were susceptible to ceftriaxone after four days of hospitalisation (412/884 [47 %] in 2012 vs. 617/1736 [36 %] in 2007 [*p* < 0.0001]) and to trimethoprim/sulphamethoxazole after three days (590/823 [64 %] in 2012 vs. 809/1787 [45 %] in 2007 [*p* < 0.0001]).

Overall, susceptibility to piperacillin/tazobactam and ceftazidime declined during this 5-year period; 1518/1973 (77 %) in 2007 vs. 727/1008 (67 %) in 2012 (*p* < 0.0001) for piperacillin/tazobactam and 1176/1975 (60 %) in 2007 vs. 492/1087 (45 %) in 2012 [*p* < 0.0001] for ceftazidime (not included in the graph). In 2012, susceptibility to piperacillin/tazobactam did not exceed 80 % after day 8 and remained below 70 % after day 12 of hospital admission. Meropenem susceptibility after 4 weeks of hospital admission was 85 % (766/896) in 2007 compared to 81 % (341/420) in 2012 (*p* = 0.047).

### Differences in antibiotic susceptibility between the two hospitals

For the four most frequently prescribed antibiotics, an analysis comparing susceptibility after one week of hospitalisation between the hospitals in 2012 was done. Ciprofloxacin and ceftriaxone susceptibility was higher in hospital A; 326/434 (75 %) vs. 263/398 (66 %); *p* = 0.004 for ciprofloxacin and 230/433 (53 %) vs. 145/398 (36 %); *p* < 0.001 for ceftriaxone. Susceptibility to amoxicillin/clavulanic acid was higher in hospital B; 117/398 (29 %) vs. 90/434 (21 %); *p* = 0.004. Susceptibility to piperacillin/tazobactam was equal in both hospitals; 271/434 (62 %) in hospital A vs. 254/396 (64 %) in hospital B; *p* = 0.610.

## Discussion

This study provides insight into the microorganisms yielded by (surveillance) cultures of ETA arranged according to the day after hospital admission and their susceptibility to frequently used antibiotics. Accordingly, possibly unconventional insights into the rationale of empirical antibiotic therapy for pneumonia in ICUs, including its appropriateness over time, are provided. Whereas the susceptibility to some frequently used antibiotics significantly and relevantly declined in the last 5 years, other antibiotics demonstrated a more steady susceptibility pattern, even after weeks of hospital admission. Overall, this study endorses the increasing problem of MDR development. In the following paragraphs, several noticeable results are presented and discussed in the light of the currently available literature.

### 2012 vs. 2007

In 2012, significantly less potentially pathogenic microorganisms (especially *P. aeruginosa*) were identified compared to 2007, which could be explained by SOD/SDD use [27] in hospital A and chlorhexidine use and the more frequent use of meropenem in hospital B (166 in 2007 vs. 367 in 2012 [expressed in daily defined dose]). Furthermore, fewer samples were obtained in 2012 compared to 2007. As *S. maltophilia* was increasingly identified, the significance of the presence of this notorious MDR microorganism [26] in the ICU remains an interesting subject for further studies.

### Early-onset vs. late-onset VAP

From an aetiological point of view, the classical early-onset pathogens were significantly less often present after four days of hospitalisation. However, the decrease in incidence of these early-onset microorganisms was less clear than perhaps expected. One may, therefore, question whether the term ‘early-onset’ is still applicable, since 45 % of the pathogens identified in the first four days of hospitalisation were not identified as early-onset pathogens. The risk that a late-onset pathogen is involved in this traditionally early-onset period is, thus, high, which may impact on the choice of empirical antibiotic therapy. On the other hand, the 45 % could be a overestimation, as *S. pneumonia* and *Legionella pneumophila* are possibly underrepresented: *Streptococcus pneumoniae* is easily killed by pre-culture administered penicillin and both bacteria might be identified by tests other than ETA cultures, such as blood culture and urine antigen testing [28].

Whereas susceptibility to amoxicillin/clavulanic acid declined similarly from 62 % on day 3 to 42 % on day 4 in 2012, this is probably irrelevant, as even 62 % susceptibility is too low. Whereas other antibiotics did not demonstrate such decline, no real cut-off could be pinpointed from the results from a treatment point of view. Indeed, recent studies support these findings and classifying VAP patients based on the time of onset does not result in the generation of two groups with different MDR rates [29] and results in under- and overtreatment [30]. Overall, the difference between early-onset and late-onset VAP is, thus, increasingly blurred and is probably irrelevant from an antibiotic treatment perspective. One wonders whether this nomenclature can be abandoned in contemporary practice, since its use appears to become obsolete.

### Empirical antibiotic appropriateness

When empiric VAP therapy is initiated irrespectively of ETA (surveillance) cultures results or knowledge of previously administered antibiotics, one may assume that the provided *antibiotic susceptibilities* could be interpreted as *appropriateness of this antibiotic* for pneumonia developing on a specific day after hospital admission. Under this assumption, amoxicillin/clavulanic acid would have been inappropriate at all times and ceftriaxone would have been only appropriate as empirical therapy on the first day of hospital admission. In 2012, piperacillin/tazobactam would have been inappropriate after day 8 of hospital admission, whereas in 2007, piperacillin/tazobactam overall appropriateness would reveal 77 %. In reality, appropriateness of the tested antibiotics is probably higher if the results of previously obtained ETA (surveillance) cultures, previously administered antibiotics and clinical features are taken into account before pneumonia treatment is started, hence accentuating their importance for pneumonia management in the ICU. Indeed, when the results of previously obtained (surveillance) cultures of ETAs are known, antibiotics will be adjusted accordingly [13, 16, 31]. Furthermore, less broad-spectrum antibiotics can likely be used when only previously yielded microorganisms are targeted [16], thereby decreasing the chances of MDR development [4] and providing good antibiotic stewardship [5].

Taking into account the costs (7 € per daily defined dose, excluding the blood drug level determination costs) and stable appropriateness level, gentamicin would appear to be the choice for empirical pneumonia treatment on ICUs. However, aminoglycosides are suboptimal for lung tissue penetration [32] and have adverse effects on auditory [33] and renal function [34, 35]. Additionally, the susceptibility of *H. influenzae*, *S. pneumoniae* and *M. catarrhalis* to gentamicin was neither tested nor known, but these microorganisms are uncommon after 1–2 weeks of hospitalisation (see Supplementary material).

Likewise, ciprofloxacin performed reasonably several weeks after hospital admission, and despite concerns about MDR development [36] and *Clostridium difficile*-associated diarrhoea [37], it may have a role as an initial empirical agent until microbiological test results are available.

### Differences between hospitals

Several differences in microorganism prevalence and antibiotic susceptibility between two neighbouring hospitals were revealed. Whereas *S. aureus*, *M. catarrhalis* and *M. morganii* were identified significantly more often in hospital B, *Acinetobacter* spp. were significantly less frequently identified in hospital B as compared to hospital A. Overall, antibiotic susceptibility was lower in hospital A, possibly due to more overall antibiotic use in the ICU of university hospitals compared to ICUs in non-university hospitals [38]. After one week of hospital admission, susceptibility to antibiotics was revealed to be significantly higher in the hospital that did infrequently incorporate that antibiotic in their empirical antibiotic protocol (hospital A ceftriaxone; hospital B amoxicillin/clavulanic acid). Susceptibility to trimethoprim/sulphamethoxazole, which are infrequently used in both hospitals, increases over the 5-year period in both hospitals. These two findings suggest that infrequently used antibiotics may be more appropriate in the future, as resistance to these antibiotics is fading away, as described previously [39, 40]. In order to prevent resistance, it could, thus, be justified to periodically adapt empirical therapy, a strategy which is called antibiotic cycling or rotation. Available studies concerning this strategy are contradictory [41–43], leaving it an interesting topic for further studies. The differences between the two hospitals emphasise the need for local microbiological surveillance and mapping for better pneumonia treatment [6, 44, 45].

### Limitations

Apart from the limitations inherent to the hypothetic model and the probable underrepresentation of *S. pneumoniae* and *L. pneumophila*, some other limitations should be addressed. First, all positive ETA culture results were included regardless of whether they were taken for surveillance or for suspected VAP. Including exclusively (suspected) VAP cases, as done in previous and partly similar research [10], would increase the strength of the study method. Yet, the high numbers of microorganisms herein identified and studied would then never have been achieved. Second, the number of readmissions, admission from nursing homes, medical history (e.g. chronic obstructive pulmonary disease, cystic fibrosis or immune state) and pre-admission culture results were not available. Lack of this information may at least partly explain the early presence of ‘late-onset’ microorganisms and could have influenced the choice of empirical therapy in actual practice, since they increase the risk of MDR microorganism involvement [6]. Third, correction for antibiotic use was not applied. When mechanically ventilated patients receive antibiotics, colonisation with resistant bacteria may occur. Fourth and last, susceptibility analyses were expressed per patient-day. As a result, patients with a prolonged hospital stay may be responsible for a superabundant number of microorganisms in the weeks far beyond hospital admission. This may have overestimated antibiotic resistance and the results of those weeks should, thus, be viewed in this perspective. Yet, a superior way to express the antibiotic susceptibility in a hospital on a given day after admission is not available. However, depending on the study design, the expression may be based on isolate, patient, episode and/or resistance phenotype [46].

## Conclusions

This study provides insights into microorganism prevalence in endotracheal aspirate (ETA) cultures in the intensive care unit (ICU) and its antibiotic susceptibility. With the method used, no purposeful cut-off could be determined to distinguish early- from late-onset ventilator-associated pneumonia (VAP) from a treatment point of view. Therefore, classifying VAP based on the time of onset has perhaps become obsolete. Amoxicillin/clavulanic acid resistance appeared high during all days of hospital admissions, whereas piperacillin/tazobactam resistance was high after eight days of hospitalisation. The decline during hospital admission in susceptibility to frequently empirically used antibiotics was more explicit in the hospital that incorporated that antibiotic in empirical treatment protocols. Overall, adapting empirical pneumonia therapy on previously known results of surveillance cultures appeared rewarding in order to increase the appropriateness of therapy.

## Electronic supplementary material

Below are the links to the electronic supplementary material.

ESM 1 (DOCX 33 kb)

ESM 2 (DOCX 37 kb)

ESM 3 (DOCX 37 kb)

ESM 4 (DOCX 37 kb)

ESM 5 (DOCX 37 kb)
