# Supplementary material for: Empirical antibiotic therapy for pneumonia in intensive care units: a multicentre, retrospective analysis of potentially pathogenic microorganisms identified by endotracheal aspirates cultures
Source: Eur J Clin Microbiol Infect Dis. 2015 Sep 18;34(11):2295–305. doi: 10.1007/s10096-015-2482-y (PMC4607706; doi:10.1007/s10096-015-2482-y)
Supplement: Supplementary file 1 — (DOCX 33 kb) [file 10096_2015_2482_MOESM1_ESM.docx]

**Appendix 1.** Corrected odds ratios including 95% confidence interval and *P* value regarding the prevalence of microorganisms identified in 2012 *vs.* 2007 and in hospital B *vs.* hospital A.

|  | 2012 *vs.* 2007 | Hospital B *vs.* hospital A |
| --- | --- | --- |
| Gram positive | 1.074 (.716-1.609) *P* = 0.731 | 2.541 (1.801-3.585) *P* < .001 * |
| *Staphylococcus aureus* | 1.061 (.694-1.620) *P* = .786 | 2.589 (1.799-3.725) *P* < .001 * |
| MRSA | 1.056 (.143-7.807) *P* = .957 | 6.148 (.709-53.316) *P* =.099 |
| *Streptococcus pneumoniae* | 1.139 (.645-2.012) *P* = .654 | 1.756 (.994-3.101) *P* = .053 |
| Gram negative | .931 (.621-1.396) *P* = .731 | .394 (.279-.555) *P* < .001 * |
| Nonfermenters | .648 (.438-.960) *P* = .03 * | .764 (.529-1.105) *P* = .153 |
| *Acinetobacter* spp. | .495 (.187-1.311) *P* = .157 | .200 (.076-.526) *P* = .001 * |
| *Moraxella catarrhalis* | .832 (.374-1.851) *P* = .652 | 4.062 (1.777-9.285) *P* = .001* |
| *Pseudomonas aeruginosa* | .470 (.262-.843) *P* = .011 * | .961 (.597-1.546) *P* = .869 |
| *Stenotrophomonas maltophilia* | 2.988 (1.458-6.126) *P* = .003* | .755 (.365-1.563) *P* = .449 |
| Other nonfermenters | 7.980 (2.152-29.596) *P* = .002 * | .276 (.07-1.089) *P* = .066 |
| *Enterobacteriaceae* | 1.357 (1.003-1.836) *P* = .047 * | .773 (.578-1.034) *P* = .083 |
| *Citrobacter* spp. | 1.289 (.575-2.886) *P* = .538 | 1.190 (.524-2.700) *P* = .678 |
| *Enterobacter* spp. | .961 (.562-1.645) *P* = .885 | .624 (.352-1.105) *P* = .106 |
| *Escherichia coli* | .978 (.652-1.465) *P* = .913 | .783 (.531-1.155) *P* = .217 |
| *Klebsiella* spp. | .851 (.563-1.286) *P* = .443 | .983 (.654-1.476) *P* = .932 |
| *Morganella morganii* | .625 (.235-1.660) *P* = .345 | 2.763 (1.120-5.816) *P* = .027 * |
| *Proteus* spp. | 1.881 (1.020-3.467) *P* = .043 * | 1.379 (.749-2.540) *P* = .303 |
| *Serratia* spp. | 2.563 (1.271-5.169) *P* = .009 * | .498 (.224-1.110) *P* = .088 |
| Other enterobacteriaceae | .717 (.209-2.459) *P* = .597 | 2.660 (.791-8.949) *P* = .114 |
| *Haemophilus influenzae* | 1.642 (1.027-2.626) *P* = .038* | 1.267 (.793-2.024) *P* = .322 |
| Other species | .796 (.060-10.488) *P* = .863 | 2.764 (.211-36.164) *P* = .438 |

* Significant difference (*P* $\leq$ .05)

All corrected Odds Ratios were calculated by Generalized Estimating Equations using IBM SPSS Statistics version 23 for Windows (Chicago, Il, USA)
