# Supplementary material for: Empirical antibiotic therapy for pneumonia in intensive care units: a multicentre, retrospective analysis of potentially pathogenic microorganisms identified by endotracheal aspirates cultures
Source: Eur J Clin Microbiol Infect Dis. 2015 Sep 18;34(11):2295–305. doi: 10.1007/s10096-015-2482-y (PMC4607706; doi:10.1007/s10096-015-2482-y)
Supplement: Supplementary file 2 — (DOCX 37 kb) [file 10096_2015_2482_MOESM2_ESM.docx]

**Appendix 2.**

Prevalence of potentially pathogenic microorganisms yielded by endotracheal aspirate samples in **2007** arranged per day for the first two weeks after hospital admission.

*S. pneumoniae*

*H. (para) influenzae*

*M. catarrhalis*

*S. aureus*

*E. coli*

*Enterobacter* spp.

*Klebsiella* spp.

*Citrobacter* spp.

*Serrratia* spp.

*M. morganii*

*Proteus* spp.

MRSA

Other *enterobacteriaceae*

Other species

*Acinetobacter* spp.

*P. aeruginosa*
